# High-Content Imaging and Machine Learning Classify Phenotypical Change in Coronary Artery Endothelial Cells Caused by BPS

**DOI:** 10.3390/ijms27073259

**Published:** 2026-04-03

**Authors:** Lavinia Eugenia Ferariu, Gheorghe Movileanu, Giulia Gaggi, Barbara Ghinassi, Angela di Baldassarre, Andrea Di Credico

**Affiliations:** 1 Department of Automatic Control and Applied Informatics, Gheorghe Asachi Technical University of Iasi, 27 Mageron, 700050 Iasi, Romania; gheorghe.movileanu@student.tuiasi.ro; 2 Department of Medicine and Ageing Sciences, G. d’Annunzio University of Chieti-Pescara, Via dei Vestini 31, 66100 Chieti, Italy; giulia.gaggi@unich.it (G.G.); a.dibaldassarre@unich.it (A.d.B.); andrea.dicredico@unich.it (A.D.C.); 3 Cell Reprogramming and Differentiation Laboratory, Center of Advanced Studies and Technologies (CAST), G. d’Annunzio University of Chieti-Pescara, 66100 Chieti, Italy; 4 UdA-Tech Laboratory, G. d’Annunzio University of Chieti-Pescara, 66100 Chieti, Italy; 5 Department of Innovative Technologies in Medicine and Dentistry, G. d’Annunzio University of Chieti-Pescara, 66100 Chieti, Italy

**Keywords:** BPS, toxicity prediction, high-content microscopy, binary classification, feature reduction

## Abstract

Bisphenol S (BPS) is widely used as a replacement for bisphenol A, yet accumulating evidence suggests that it has comparable endocrine and cardiovascular toxicity. Here, we investigated whether prolonged low-dose BPS exposure induces subtle but classifiable phenotypic alterations in human coronary artery endothelial cells (HCAEC), using an end-to-end experimental and ML pipeline that spans cell culture, high-content imaging, feature extraction, and robust classification. Cells were exposed to 0.1 µM BPS for 96 h and profiled using a cell painting assay and high-content microscopy. Image segmentation yielded ~2500 quantitative features per cell across four compartments—*Membrane*, *Cytoplasm*, *Ring* region (i.e., perinuclear region), and *Nucleus*—for multiple fluorophores. We systematically compared different classifiers (Random Forest, XGBoost, LASSO logistic regressor) using feature selection (MRMR, ReliefF, LASSO) or transformation-based dimensionality reduction (PCA, autoencoders). Tree-based ensembles robustly handled high-dimensional inputs, with XGBoost combined with ReliefF-selected features achieving the best performance. The most informative descriptors predominantly mapped to mitochondrial and nuclear channels, indicating early alterations in mitochondrial organisation and chromatin-related features. These findings show that chronic low-dose BPS exposure elicits a distinct endothelial phenotype, consistent with early endothelial dysfunction, and demonstrate that integrating high-content imaging with machine learning provides a sensitive, scalable framework for vascular toxicity assessment of environmental contaminants.

## 1. Introduction

Bisphenol S (BPS) is a synthetic compound widely used as a substitute for bisphenol A (BPA) in the production of plastics, resins, and thermal papers. Although initially introduced as a safer alternative, accumulating evidence indicates that BPS, like BPA, can act as an endocrine-disrupting chemical (EDC) with adverse effects on human health [1,2]. Importantly, BPS and other EDCs can cross the placental barrier, causing adverse effects on both the exposed population and their offspring [3,4]. One of the most concerning aspects of bisphenol exposure is its potential impact on the cardiovascular system, with accumulating evidence that the hormonal milieu modulates cardiovascular susceptibility [5,6,7]. Endothelial cells, which line the inner surface of blood vessels, play a central role in vascular homeostasis, regulating barrier function, angiogenesis, and vascular tone. Perturbations of endothelial morphology and function are strongly associated with pathological processes such as atherosclerosis, hypertension, and metabolic syndrome [8,9].

The coronary artery endothelium represents a highly relevant cellular model for toxicity testing. As the vessels that supply blood to the myocardium, coronary arteries are continuously exposed to hemodynamic stress and to circulating toxicants absorbed from the environment. Even subtle morphological or functional alterations in coronary artery endothelial cells may predispose to vascular inflammation, lipid accumulation, and plaque formation, key early events in atherosclerosis and cardiovascular disease [10,11]. Studying the response of coronary artery endothelial cells to BPS is therefore crucial, as it provides direct insight into the potential contribution of this compound to vascular dysfunction and cardiovascular risk.

In this study, we specifically address the challenge of detecting and characterising the subtle cellular alterations induced by prolonged exposure to BPS in human coronary artery endothelial cells. Conventional toxicological approaches often rely on targeted assays measuring a limited number of biochemical or morphological endpoints, which may fail to capture the complex and heterogeneous nature of cellular responses. High-content imaging (HCI) combined with high-throughput methods, such as cell painting, provides an opportunity to extract thousands of quantitative features from multiple cellular compartments and fluorescent stains, offering a holistic description of cell state and morphology [12,13]. However, the sheer dimensionality and redundancy of these datasets make manual analysis impractical and demand the integration of automated computational methods.

Machine learning (ML) has emerged as a powerful approach for identifying relevant features, reducing dimensionality, and building predictive models in complex biological datasets [14]. By combining HCI with ML algorithms, it becomes possible to classify cellular phenotypes and predict toxicity outcomes with high accuracy. In this regard, in a recent study the effect of several bisphenols and perfluoroalkyles was tested on human midbrain dopaminergic neurons using HCI and ML; the results showed that the combination of these two methods led to a high classification accuracy of the different cellular phenotypes based on the type of treatment, also revealing subtle changes that would have probably not been detected with more basic analyses [15]. Despite this potential, a few studies have systematically applied such approaches to investigate BPS-induced vascular toxicity, leaving a significant gap in the field of computational toxicology.

This work aims to develop and validate a multidisciplinary pipeline that integrates high-content imaging and machine learning to predict the cellular toxicity of BPS exposure. By comparing multiple dimensionality reduction strategies and classifiers, we identify robust models capable of distinguishing BPS-treated from control endothelial cells with high accuracy. The dual perspective, integrating computational assessment with biological interpretation, enables a refined analysis of BPS-induced phenotypic signatures. Specifically, our main contributions include:

- An experimental workflow that combines controlled low-dose BPS exposure of primary endothelial cell cultures with high-content imaging for single-cell feature extraction.
- A detailed feature-relevance analysis across cellular compartments and fluorophores, providing biologically interpretable insights into how BPS perturbs specific subcellular structures.
- A systematic comparison of dimensionality-reduction strategies, including both feature-selection and feature-transformation approaches (e.g., PCA and autoencoder-based embeddings), applied to the full feature set and to disjoint subsets of attributes.
- A comparative evaluation of computationally efficient classifiers integrated with feature-reduction techniques into a unified pipeline for the detection of BPS-induced toxicity.

## 2. Results

### 2.1. Feature Selection

The analysis of cell painting by means of Harmony Software version 5.2provides a large set of features extracted from different cellular regions after segmentation (*Nucleus*, *Ring region*—representing perinuclear space, *Cytoplasm*, and *Membrane*) across multiple fluorophores (*PhenoVue Hoechst 33342 Nuclear Stain*, *PhenoVue Fluor 488 Concanavalin A*, *PhenoVue 512 Nucleic Acid Stain*, *PhenoVue Fluor 555 WGA*, and *PhenoVue 641 Mitochondrial Stain*). Consequently, the data analysis first focused on evaluating feature relevance and redundancy as a preliminary step toward dimensionality reduction. These aspects were assessed using mutual information (MI) and the Minimum Redundancy Maximum Relevance (MRMR) algorithm. In addition, the ReliefF algorithm was applied to explore contextual dependencies within the feature set, while linear regression with Least Absolute Shrinkage and Selection Operator (LASSO) was used to evaluate potential linear dependencies. Lastly, the Out-of-Bag (OOB) ranking method was employed to analyse feature importance in a pretrained ensemble of trees by measuring the performance impact of randomly permuted feature values.

**Figure 1:**
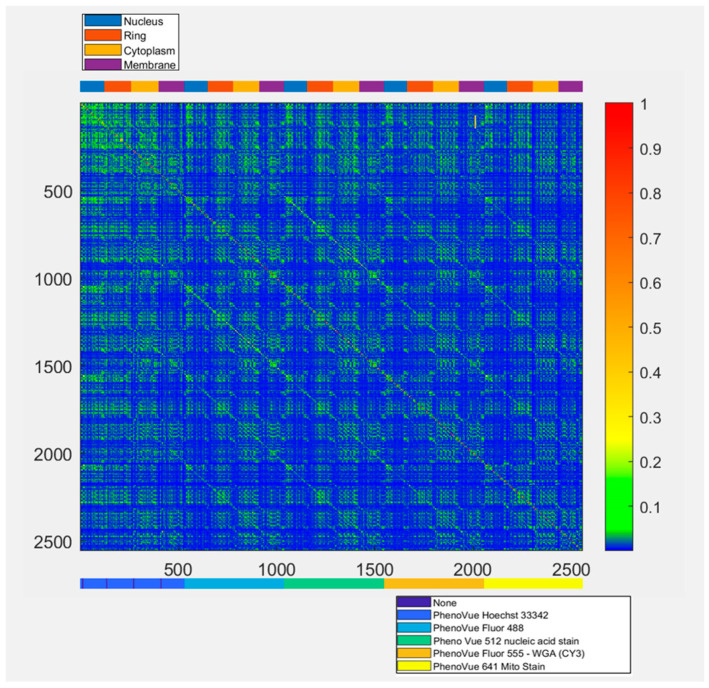
Feature redundancy assessed via MI between feature pairs (large values for dependent features); the values are scaled in [0, 1]. The top colour bar indicates the cellular region associated with each feature, while the bottom bar denotes the corresponding fluorescent dye. X and Y axis labels represent feature indices.

Figure 1 shows that the MI values for feature pairs are generally low, even within the same cell region, indicating limited redundancy. These findings suggest that effective feature reduction may be challenging, even if MI is limited to evaluating pairwise dependencies and does not capture interactions among multiple features.

**Figure 2:**
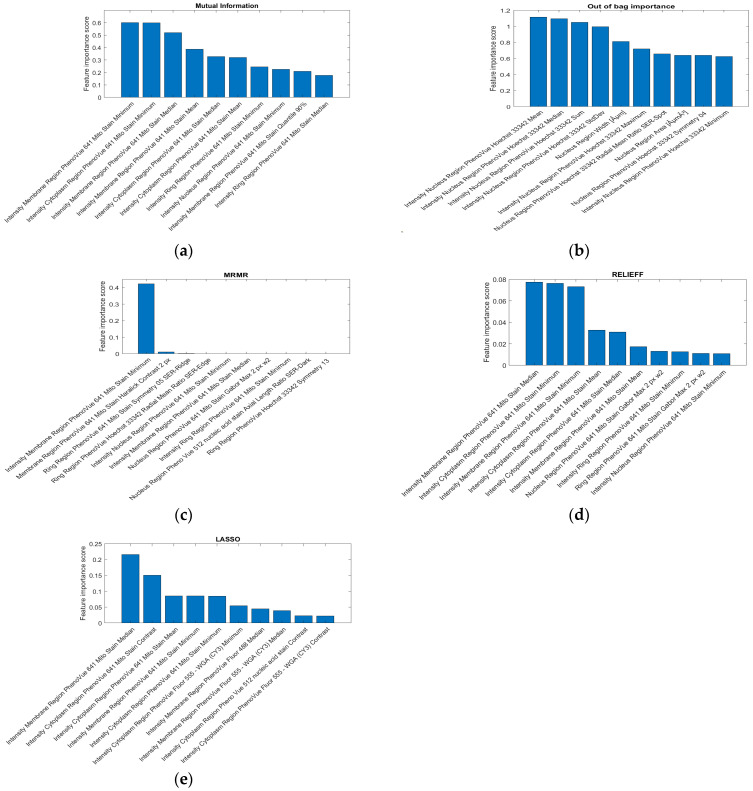
Top 10 features ranked by importance using different algorithms: (**a**) MI between features and target class. (**b**) OOB-based ranking. (**c**) MRMR. (**d**) ReliefF. (**e**) LASSO logistic regression.

**Figure 3:**
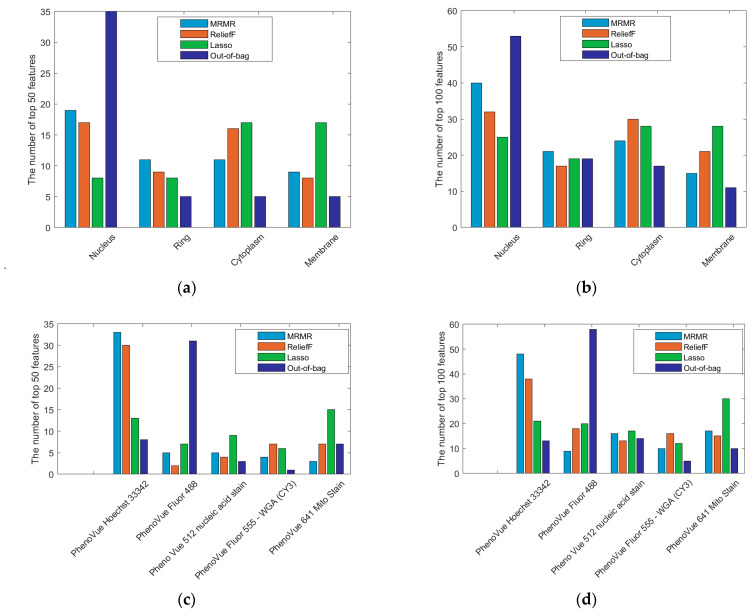
Histograms of the most informative features across cell regions and fluorescent stains, selected using MRMR, ReliefF, LASSO and OOB-based ranking: (**a**) Top 50 features distributed across cell regions. (**b**) Top 100 features distributed across cell regions. (**c**) Top 50 features distributed across fluorophores. (**d**) Top 100 features distributed across fluorophores.

For all the aforementioned algorithms, the top 10 features ranked by relevance to the classification task are shown in Figure 2, while Figure 3 presents histograms of the top 50 and 100 most informative features, grouped by cell region and fluorescent dye. Although MI, MRMR, ReliefF, and LASSO apply different strategies to assess feature relevance—leading to some variability—the majority of the top 10 features identified by these methods are associated with the *PhenoVue 641 Mito Stain*. This preference is also reflected in the top 50 and 100 features selected by MRMR and ReliefF, which can effectively capture nonlinear dependencies among features. In contrast, the OOB ranking yields a markedly different hierarchy, emphasising features related to *PhenoVue Hoechst 33342*. Regarding cell regions, both MRMR and OOB ranking tend to favour features from the *Nucleus*, although OOB selects a few features outside this region. ReliefF, however, highlights the *Cytoplasm* and the *Nucleus*, while LASSO emphasises both the *Cytoplasm* and the *Membrane*.

As detailed in the next subsection, MRMR and ReliefF proved to be effective feature reduction methods, producing models with high accuracy on both training and testing sets, thereby validating the value of the feature hierarchies they generate.

The hierarchy obtained from OOB ranking shows big differences compared to MRMR and ReliefF. These differences can arise because OOB importance is influenced by correlations among features: when two variables integrate overlapping information, permuting one may show little effect if the other can compensate.

These disagreements between feature selection methods suggest that aggressive dimensionality reduction may lead to the loss of important input information. To address this, we employed classification algorithms that effectively manage high-dimensional inputs and are inherently robust to data redundancy, such as tree-based ensembles. Additionally, hyperparameter tuning was performed via grid search, applied to both the feature reduction and classification steps within a unified pipeline, ensuring consistent and relevant evaluation.

**Figure 4:**
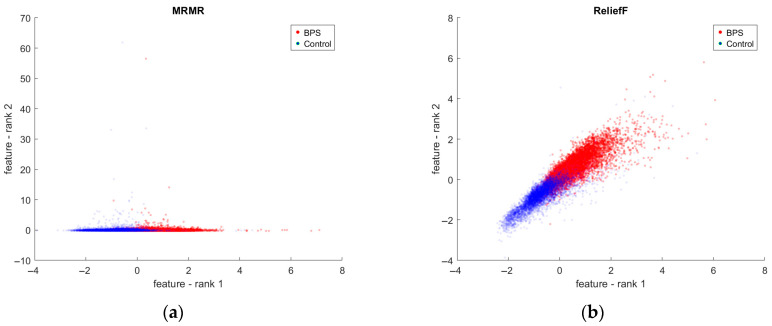
The distribution across classes according to the two most informative features recommended by the feature selection algorithms: (**a**) MRMR (feature—rank 1: *Intensity Membrane Region PhenoVue 641 Mito Stain Minimum*, feature—rank 2: *Membrane Region PhenoVue 641 Mito Stain Haralick Contrast 2ptx*). (**b**) ReliefF (feature—rank 1: *Intensity Membrane Region PhenoVue 641 Mito Stain Median*, feature—rank 2: *Intensity Cytoplasm Region PhenoVue 641 Mito Stain Minimum*).

Figure 4 shows the distribution of data points across the two classes, based on the two most informative features identified by MRMR and ReliefF. Although these features alone do not provide a complete separation of the classes, they still enable the correct classification of many examples.

**Figure 5:**
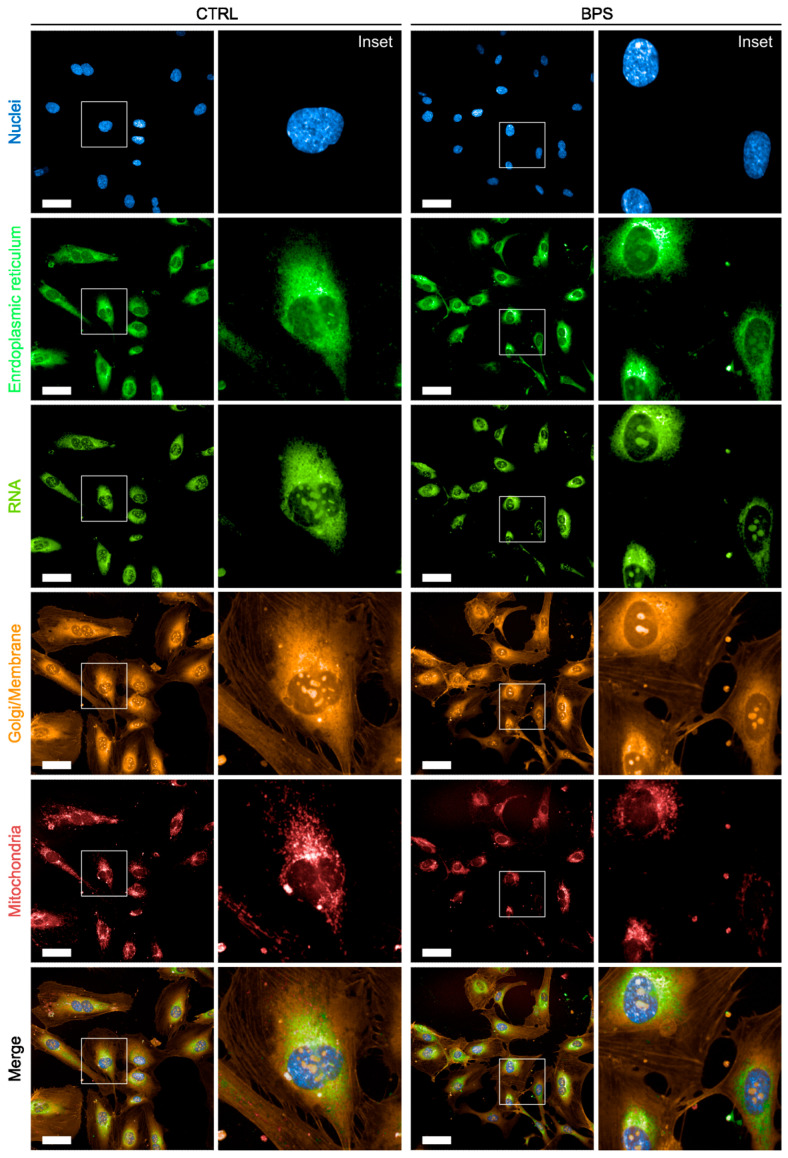
Representative high-content microscopy images of human coronary artery endothelial cells (HCAEC) exposed to vehicle control (CTRL) or 0.1 µM Bisphenol S (BPS) for 96 h and stained using the PhenoVue Cell Painting assay. For each condition, a representative field acquired at 40× magnification and a higher-magnification inset are shown. Rows correspond to the individual fluorescence channels: *Hoechst 33342* (nuclei), *PhenoVue Fluor 488 Concanavalin A* (endoplasmic reticulum and intracellular membranes), *PhenoVue 512 nucleic acid stain* (RNA/nucleoli), *PhenoVue Fluor 555* wheat germ agglutinin (plasma membrane), *PhenoVue 641 mitochondrial stain* (mitochondria), and the merged image. White boxes represent the part of the image used for the related inset. Scale bar: 50 µm, 40× objective.

Consistent with the feature importance rankings obtained using MRMR and ReliefF, Figure 5 shows that the most visually informative differences are observed in the mitochondrial channel. In BPS-treated cells, the mitochondrial signal displays decreased mean intensity, increased heterogeneity in intensity and spatial organisation, with localised high-intensity regions and a tendency toward perinuclear clustering. These visual patterns correspond to the predominance of mitochondrial intensity- and texture-related features—particularly median and minimum intensity values and Haralick contrast descriptors—that emerged as the most informative variables driving classification performance.

In contrast, nuclear features stained with Hoechst 33342 contribute to the classification primarily through changes in intensity distribution and texture rather than gross morphological alterations. In the images, BPS-treated cells show increased intranuclear variability and less uniform chromatin-associated signal, consistent with the selection of nuclear texture and intensity descriptors among the extended sets of informative features identified by the ML pipeline. Importantly, no clear signs of nuclear fragmentation or size reduction are observed, in agreement with the absence of overt cytotoxicity.

Cytoplasmic and membrane-associated channels, visualised by Concanavalin A, the 512 nucleic acid stain, and WGA, do not individually exhibit a big qualitative difference, but display increased cell-to-cell variability in BPS-treated samples. This observation aligns with the behaviour of ReliefF-based feature selection, which captures local and contextual differences between neighbouring samples and benefits from heterogeneous, population-level variations rather than uniform shifts.

Overall, the visual phenotype observed in the images shows that chronic low-dose BPS exposure induces a subtle but classifiable endothelial phenotype dominated by mitochondrial reorganisation and complemented by nuclear and cytoplasmic heterogeneity, rather than by overt structural damage.

### 2.2. Feature Reduction and Classification

The experimental setup is summarised in Table 1, and selected results for the configurations explored via end-to-end grid search are presented in Table 2, Table 3 and Table 4. Tree-based ensemble models—specifically Random Forest (RF) and Extreme Gradient Boosting (XGBoost)—were chosen for their robustness in handling high-dimensional inputs that may include irrelevant or redundant features. To complement these models, we also employed LASSO logistic regression, valued for the interpretable and sparse representations it produces. All models provide a good balance between explainability, computational efficiency and classification performance.

Following common recommendations for classification tasks with numerous attributes, we also considered a pipeline that starts with a feature reduction to generate compact vectors, thereby simplifying the task of classification models. This reduction was achieved through feature selection using MRMR, ReliefF, or LASSO. Additionally, we examined transformation-based reduction methods such as Principal Component Analysis (PCA) and autoencoders (AEs), applied either to the complete feature set or to disjoint subsets, to alleviate the computational challenges posed by high-dimensional data. The reduced feature set or the full original set was then used for binary classification.

Both Random Forest and XGBoost achieved good accuracy when applied to the full feature vector, but performance improved in some configurations using reduced feature sets selected with MRMR or ReliefF. In contrast, LASSO and PCA yielded poorer results in feature reduction, suggesting that their reliance on linear dependencies oversimplifies relationships in this dataset. However, PCA—by exploiting the training covariance matrix—performed better than LASSO. PCA and autoencoders produced reasonable results only when a large number of features were retained after dimensionality reduction. By effectively capturing complex nonlinear dependencies, autoencoders outperformed PCA, retaining fewer but more informative features, particularly when applied to feature subsets. In such cases, each autoencoder had few learnable parameters, enabling efficient independent training.

Using boosting techniques to configure the subset of samples for each new tree, XGBoost offered slightly better results than Random Forest. Without a hard limitation on the tree depth, even ensembles with relatively few trees achieved good performance, with the most influential factor remaining the content and size of the feature vector passed to the classifier. This aspect is important for the explainability, offering premises for the interpretation of results.

The highest classification accuracy was obtained with XGBoost combined with ReliefF for feature selection, achieving 100% on training and 95.63% on testing (configuration II-42 in Table 2).

## 3. Discussion

The present study demonstrates that prolonged exposure to BPS induces measurable phenotypic changes in human coronary artery endothelial cells, which can be detected using high-content microscopy combined with advanced machine learning approaches. Even if BPS was introduced as a safer alternative to Bisphenol A (BPA), our findings align with mounting evidence that it retains endocrine-disrupting properties and may contribute to cardiovascular risk [1,16]. Endothelial dysfunction is widely recognised as a precursor of atherosclerosis, hypertension, and metabolic syndrome [17], and the coronary endothelium is especially vulnerable due to its constant exposure to circulating xenobiotics and hemodynamic stress. Thus, even subtle BPS-induced morphological or functional changes may have clinical implications by predisposing to vascular inflammation and plaque formation.

The contributions of this study are twofold: (i) we provide a methodological framework for leveraging imaging-based feature-rich datasets in toxicity assessment, and (ii) we deliver new biological insights into the adverse cellular effects of BPS on human coronary endothelium.

Although previous studies have explored the cellular effects of bisphenol analogues using imaging or machine-learning tools, our work presents some methodological advancements. First, we perform feature-level relevance analysis at subcellular resolution, distinguishing contributions from specific compartments and fluorophores. Second, we conduct an extensive comparison of dimensionality-reduction strategies, including PCA and autoencoders operating on feature subsets, enabling a refined understanding of the role that different features play in classification. Third, we analyse a broad panel of classifiers, ranging from tree-based models to sparse linear Lasso regression, within a unified pipeline. Together, these elements provide a comprehensive ML framework, supporting a robust evaluation of phenotypic alterations induced by low-dose BPS exposure.

The high predictive accuracy obtained with tree-based ensemble methods, particularly XGBoost (training: 100%, testing: 95.6%), has important implications for toxicity detection. These results demonstrate that ML models trained on high-content microscopy data can reliably distinguish between control and BPS-treated cells, even when morphological changes are subtle and heterogeneous. This sensitivity exceeds the capability of conventional targeted assays, which often measure only a handful of endpoints. Similar to recent studies using HCI + ML pipelines in neuronal toxicity assays [15], our results confirm that unbiased feature extraction combined with robust classifiers can capture complex cellular phenotypes and uncover toxicant effects that might otherwise remain hidden.

From a biological perspective, the fact that BPS-treated cells can be robustly classified despite the absence of overt cytotoxicity suggests that BPS primarily induces a “pre-lesional” endothelial phenotype, characterised by subtle remodelling of organelle function rather than gross cell damage. Endothelial cells are exquisitely sensitive to changes in redox balance, mitochondrial homeostasis and chromatin organisation, and early alterations in these compartments are increasingly recognised as determinants of endothelial dysfunction and cardiovascular disease [18]. In this context, the phenotypic fingerprints captured by our pipeline likely represent an integrated readout of early stress responses that precede the classical hallmarks of vascular pathology.

The comparison of feature reduction algorithms revealed discrepancies across methods employing different techniques, highlighting the inherent challenges of high-dimensional data analysis. To address this, integrating feature reduction and classification within the pipeline proved essential for additional validation. Both MRMR and ReliefF consistently yielded compact, informative feature subsets that improved classification while preserving discriminatory power. In contrast, LASSO and PCA, though valuable for interpretability, tended to oversimplify nonlinear relationships, leading to diminished performance. Autoencoders offered a promising alternative by capturing nonlinear dependencies more effectively than PCA; however, their design poses challenges when working with the full feature vector at once. Overall, these findings support previous reports that nonlinear feature reduction is critical in cellular imaging studies, where toxicity phenotypes are strongly context-dependent [19]. Working with disjoint feature subsets was advantageous only for autoencoders, as it enabled the use of models with fewer learnable parameters.

A performance–interpretability trade-off emerges from the design of the feature reduction and classification steps. Models based on fewer features are easier to interpret, whereas configurations retaining larger feature sets achieve higher accuracy but reduce transparency. Nevertheless, all classifiers employed in this study remained computationally lightweight, ensuring that the overall pipeline is not resource-intensive despite the moderate increase in model complexity.

According to MRMR and ReliefF, which offered the best classification results, the majority of the top 10 relevant features corresponded to *Region PhenoVue 641 Mito Stain* and primarily reflected intensity rather than shape (Figure 2c,d). However, this outcome may also be influenced by the performance of the segmentation algorithms, which can alter cellular region shapes and consequently affect shape-related features. Both MRMR and ReliefF suggest that the most significant effects of BPS are reflected in the intensity of the *Membrane*. Although the ranking position of these descriptors cannot be guaranteed, their relevance is further highlighted by Figure 4, which demonstrates strong discriminative power between the two classes when using only the top two features ranked by MRMR and ReliefF, respectively. For MRMR, the second feature illustrating other potentially important effects of BPS captures the Haralick contrast across the *Membrane*, whereas for ReliefF, the second feature corresponds to intensity variations across the *Cytoplasm*.

The predominance of mitochondrial features among the most informative descriptors strongly suggests that BPS exposure perturbs mitochondrial organisation or function in the coronary endothelium. Changes in mitochondrial stain intensity and texture at the membrane and cytoplasmic regions are compatible with alterations in mitochondrial mass, distribution, or membrane potential, all of which are central regulators of endothelial nitric oxide (NO) production, calcium handling and redox signalling [20,21,22]. This interpretation is consistent with mechanistic studies in human umbilical vein endothelial cells, where BPS has been shown to increase mitochondrial reactive oxygen species, depolarise the mitochondrial membrane and reduce NO bioavailability, ultimately leading to endothelial dysfunction [23,24,25]. Accordingly, in male hiPSCs, BPS exposure is associated with alterations in OXPHOS proteins, supporting the idea that mitochondria are an early hub of response to EDCs [26]. Because mitochondrial dysfunction is a recognised driver of atherosclerosis and coronary microvascular disease [27,28,29], the mitochondrial-centric signature identified here provides a plausible link between low-dose BPS exposure and early coronary vascular injury.

An overly aggressive feature reduction altered classification performance, showing that many features play an important role in BPS detection. For example, Table 2 shows a notable improvement in test-set accuracy of XGBoost with 150 trees when the number of selected features increases from 3 to 100: accuracy increases from 88.66% to 94.87% for MRMR (I-27 vs. I-33), and from 88.91% to 95.63% for ReliefF (I-36 vs. I-42). An intuitive summary across the top 50 and 100 features is illustrated in Figure 3 to highlight the regions and fluorophores with the largest number of relevant attributes. Interestingly, although many of the top 10 features identified by MRMR and ReliefF are associated with *Region PhenoVue 641 Mito Stain*, Figure 3 reveals numerous other relevant features linked to *PhenoVue Hoechst 33342*. The regions contributing the largest number of selected features are the *Nucleus* for MRMR, and both the *Nucleus* and *Cytoplasm* for ReliefF, suggesting that modifications in these regions are particularly informative for BPS detection. Notably, the top 50 and top 100 feature sets include attributes from all regions and dyes, indicating that accurate classification relies on integrating information across multiple perspectives. This observation holds across all feature selection algorithms, although they may show a preference for regions and fluorophores different from those highlighted by MRMR and ReliefF.

The contribution of nuclear and cytoplasmic Hoechst-based features further supports the notion that BPS elicits coordinated alterations in chromatin state and global transcriptional activity. Nuclear intensity and texture descriptors are frequently associated with changes in chromatin condensation, DNA damage responses, or cell-cycle distribution, all of which have been reported in endothelial and other cell types exposed to bisphenols and related endocrine disruptors [30]. Interestingly, it was demonstrated that BPs mixed with other EDCs impact epigenetic regulators [31]. Therefore, the nuclear texture shift shown in the present investigation is biologically consistent as an early signal of the alteration caused by BPS.

In endothelial cells, epigenetic and transcriptional reprogramming are integral components of the transition from a quiescent to an activated, pro-inflammatory phenotype that favours leukocyte adhesion and plaque initiation [32]. Thus, the multi-compartment signature uncovered by our analysis, spanning mitochondria, nucleus and cytoplasm, is consistent with a model in which chronic low-dose BPS promotes a shift towards an activated, “at-risk” coronary endothelial state, even in the absence of overt cell death.

Our results extend earlier findings in both scope and methodology. Previous studies on BPS toxicity have largely focused on endocrine disruption, reproductive outcomes, or neuronal effects [33,34]. Few studies have investigated vascular cells, and even fewer have applied systematic computational toxicology approaches. Compared to studies on dopaminergic neurons [15], our work demonstrates that endothelial cells show similarly subtle but classifiable phenotypic signatures under BPS exposure. This not only reinforces the validity of combining HCI with ML [35] but also expands its application to cardiovascular toxicology, a field where sensitive predictive assays remain limited.

Importantly, our findings in human coronary artery endothelial cells complement recent functional and mechanistic studies, showing that BPS impairs endothelial-dependent vasodilation, increases oxidative stress, and disrupts NO signalling in umbilical vein endothelial cells and mouse resistance arteries, as well as in developmental and prenatal exposure models [36]. Together with in vivo data demonstrating that BPS alters cardiac function and increases cardiovascular risk markers in rodents [37], these converging lines of evidence support the classification of BPS as a bona fide cardiovascular toxicant rather than a neutral BPA replacement. The fact that we detect consistent phenotypic alterations at 0.1 µM, a concentration in the range of reported human biomonitoring levels, reinforces the concern that chronic environmental exposure could contribute to coronary microvascular dysfunction in bisphenol-exposed populations [38].

Despite the strong classification performance, some limitations of the approach warrant attention, arising both from the characteristics of the dataset and from key stages of the machine-learning processing workflow:

- The dataset originates from a single-dose exposure applied to a single lot of primary endothelial cells. While this design limits the exploration of dose–response relationships and broader biological variability, the experiment still captures substantial phenotypic diversity: cells were imaged across multiple wells, 25 fields per well, and three biological replicates per condition. Moreover, the selected concentration reflects commonly reported human exposure levels to BPS, thereby supporting the biological relevance of the investigation. These treatment conditions ensure that the variability in raw data is mainly related to differences across cells.
- Feature extraction relies on segmentation accuracy, which may alter the reliability of some descriptors. However, the use of a high-resolution microscope and a robust imaging acquisition system ensures high segmentation accuracy, which reduces the risk of feature extraction errors.
- Different reduction methods may produce divergent results, and establishing clear links between selected attributes and concrete biological mechanisms remains a significant challenge [39]. However, the integrated pipeline enables a direct assessment of their quality through their effect on final classification performance. In addition, we deliberately avoided excessive dimensionality reduction, given that the classifiers employed are inherently robust to redundant features.
- While the ML models perform robustly within the current dataset, their generalizability to other imaging platforms, cell types, or experimental conditions remains to be validated. This limitation persists despite the extensive exploration of multiple classifiers and feature-reduction settings, as model performance may still depend on dataset-specific characteristics.
- Although the pipeline was designed to enhance interpretability through feature-selection strategies and the use of interpretable models (such as tree-based ensembles or sparse linear classifiers), the explainability of the best-performing configuration remains limited. The top model relies on 500 selected features and an XGBoost classifier with 150 trees, which, despite its strong predictive performance, reduces the transparency of the decision-making process.

Future work should integrate validation through extended targeted experiments. In particular, our results motivate dedicated mechanistic studies focused on mitochondrial and nuclear endpoints in the coronary endothelium. Follow-up experiments could quantitatively assess mitochondrial membrane potential, respiratory capacity, and mitochondrial ROS production using targeted high-content assays [40], coupled with measurements of NO bioavailability and eNOS coupling status, as previously described for BPS-treated HUVECs [36]. Parallel transcriptomic and epigenomic profiling would help to clarify whether the nuclear features identified here reflect activation of oxidative stress, inflammatory and endothelial activation pathways similar to those reported for BPA and its analogues [41]. By anchoring the image-derived phenotypes to defined molecular pathways, such studies would increase the mechanistic interpretability of our pipeline and further support the use of cell painting as an early warning tool for vascular toxicants.

The present findings open several avenues for future research. First, mechanistic validation should accompany phenotypic profiling, using complementary assays such as transcriptomics, metabolomics, and mitochondrial functional tests to confirm the biological pathways disrupted by BPS. Second, broader datasets encompassing multiple endothelial and vascular cell types, as well as organotypic and iPSC-derived vascular models, could provide more physiologically relevant insights. Third, dose–response and chronicity studies are needed to connect in vitro findings to real-world human exposures. Finally, epidemiological studies linking environmental BPS exposure to cardiovascular outcomes would provide essential translational context.

## 4. Materials and Methods

### 4.1. Cell Culture and Maintenance

Primary human coronary artery endothelial cells (HCAEC; ATCC^®^ PCS-100-020™, Innovation, VA, USA) were cultured according to the supplier’s recommendations. Cells were maintained in Vascular Cell Basal Medium (ATCC^®^ PCS-100-030™, USA) supplemented with the Endothelial Cell Growth Kit-VEGF (ATCC^®^ PCS-100-041™, USA), yielding a final medium containing 2% foetal bovine serum, recombinant human VEGF, EGF, FGF-basic, IGF-1, heparin sulfate, hydrocortisone hemisuccinate, L-glutamine, and ascorbic acid. Cultures were incubated at 37 °C in a humidified atmosphere containing 5% CO_2_ and subcultured at approximately 80% confluence using Trypsin-EDTA for Primary Cells (0.05% trypsin, 0.02% EDTA; ATCC^®^ PCS-999-003™, USA), followed by neutralisation with Trypsin Neutralising Solution (ATCC^®^ PCS-999-004™, USA). Cells were fed every 48 h with fresh complete medium. All experiments were conducted with cells between passages 3 and 6.

### 4.2. Cell Treatment, Cell Painting and High-Content Microscopy

HCAEC were seeded into 96-well Phenovue plates (Revvity, Waltham, MA, USA) at a density of 1 × 10^3^ cells/cm^2^ in complete endothelial growth medium and allowed to adhere and grow for 48 h (37 °C, 5% CO_2_). After this initial period, cells were treated with either 0.1 µM BPS (diluted in culture medium) or the corresponding methanol vehicle control (final methanol concentration ≤ 0.1%) for 96 h under standard culture conditions. At the end of the treatment, cells were fixed, permeabilized and stained using the *PhenoVue Cell Painting Kit* (Revvity, USA) according to the manufacturer’s instructions: briefly, cells were washed once in PBS, fixed in 4% paraformaldehyde for 20 min at room temperature, permeabilized in 0.1% Triton X-100 for 10 min, and stained with the six-probe mixture (*Hoechst 33342*, *Fluor 488 Concanavalin A*, *512 Nucleic Acid Stain*, *WGA Fluor 555*, *Mitochondrial Stain 641*) in the supplied diluent. Staining was carried out for 30 min at 37 °C in the dark, then wells were washed three times in PBS and kept in PBS for imaging. Plates were imaged on an Operetta CLS high-content imaging system (Revvity) using a 20× water-immersion objective, acquiring 25 fields per well in each fluorescence channel. Image segmentation and feature extraction were performed in Harmony software version 5.2 (Revvity) using the dedicated “Cell Painting” building-block workflow to extract morphological, intensity and texture features per cell. Cells were segmented, and the following compartments were considered: *Membrane*, *Nucleus*, *Cytoplasm*, and *Ring* region (i.e., perinuclear zone). Statistical values computed per cell, such as mean and standard deviation, were then used for downstream multivariate phenotypic profiling. All treatments and staining were performed in biological triplicate per condition.

In this study, the variability in the data is driven primarily by intrinsic cell-to-cell differences rather than by the treatment itself. Our aim was not to investigate dose-dependence but to reliably detect toxicity, and for this purpose, capturing natural variability across all available cells was important. We chose to use a single concentration and a fixed exposure time for both biological and methodological reasons. The chosen dose falls within the range of environmentally relevant human exposure reported in biomonitoring studies, making our model directly translatable to real-world conditions. Moreover, low micromolar and sub-micromolar bisphenol levels are typically used to mimic chronic environmental exposure rather than acute toxicity. Our experimental design specifically aimed to capture subtle, early, non-cytotoxic phenotypic alterations induced by BPS. Using a single, physiologically relevant low dose minimises confounding effects associated with high-dose cytotoxicity and allows us to detect early “pre-lesional” endothelial changes. Consistent with this rationale, we observed no overt cell death or morphological damage, but a distinct and classifiable phenotypic signature emerged.

### 4.3. Preparing the Training and Testing Datasets

The binary classification problem addressed in this study involves a large set of numerical features, which introduces a significant risk of redundancy. The design workflow was specifically tailored to mitigate this issue, as detailed in the following subsections. The implementation was carried out in MATLAB 2024b, with Python 3.11.9 interfacing for XGBoost.

**Figure 6:**
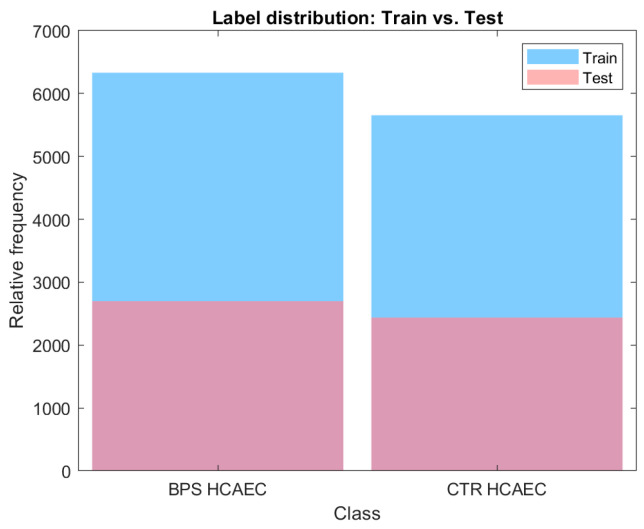
Data distribution across classes for the training and testing datasets.

A total of 17,118 samples, corresponding to cells in either normal conditions or exposed to BPS, were randomly divided into training (70%) and testing (30%) subsets, with class stratification performed to ensure balanced class distribution in both sets (Figure 6). We adopted a holdout strategy to keep the test set fully separated from all model design steps, avoiding the risk of data leakage. More specifically, we performed all feature selection, dimensionality reduction, and model tuning strictly on the training set. Grid search operated only within this portion, ensuring no test information influenced feature relevance or model optimisation. The test set was used only at the end to evaluate the final models using the already fitted transformations. Given the large dataset, this approach provides ample training data while ensuring an unbiased evaluation on unseen samples.

The first preprocessing step involved mean-based imputation to replace missing values. An analysis of missing data led to the elimination of 18 attributes with no valid values, all originating from the *Membrane* region. Additionally, 7 attributes were removed due to negligible variance, defined as $std\left(X_{i}\right)\;<\;min(\frac{mean\left(X_{i}\right)}{10},0.01$), where $std(X_{i})$ and ${mean(X}_{i})$ denote the standard deviation and mean of the feature $X_{i}$, respectively. Most of these attributes were extracted from the *Membrane* and *Cytoplasm* regions using Gabor filtering. The remaining 2557 attributes were zero-centred via normalisation. These preprocessing steps were also applied to the testing set, using information derived from the training data, including feature means, standard deviations, and the indices of removed columns.

Feature redundancy and relevance were examined using MI. Additionally, to obtain a robust evaluation of feature importance and reduce biases associated with individual methods, multiple techniques were employed: MRMR, ReliefF, LASSO, and OOB ranking. MRMR, ReliefF, and LASSO are described in the next subsection and were also used for feature selection. The OOB ranking was applied to a Random Forest model with 150 trees, trained on the full feature set without data reduction (configuration I-3 in Table 2). For each tree, the out-of-bag samples ignored during training were identified, and the increase in prediction error after random permutations of feature values was evaluated; features whose permutation caused the largest performance drop were considered the most important.

### 4.4. Feature Reduction

Eliminating redundant and irrelevant features is a crucial step in designing effective classification systems. Although the adopted models offer robustness to such features and maintain a reasonable level of interpretability, reducing the input dimensionality can still significantly enhance their performance. To evaluate this, we compared feature selection and transformation-based approaches, assessing their ability to generate compact and informative feature vectors for the binary classification task.

#### 4.4.1. Feature Selection

Feature selection is preferred because it directly identifies the most relevant features from the original set, without applying transformations, thereby preserving their inherent interpretability. To this end, we investigated a range of methods, each relying on distinct criteria to assess feature importance.

- The MRMR algorithm evaluates feature importance by leveraging mutual information: it measures relevance through the mutual information between each feature and the target class, and redundancy through the mutual information between pairs of features. Beyond its use in MRMR-based feature ranking, mutual information was also applied to separately evaluate feature importance and redundancy across distinct cell regions and fluorescent dyes. Although MI and MRMR do not explore interactions among multiple features, they are powerful techniques for highlighting complex dependencies among data.
- For randomly selected samples, the ReliefF algorithm analyses the distances along each feature to the k nearest neighbours from the same class and from different classes, based on the premise that informative features exhibit high intra-class similarity and large inter-class dissimilarity. Given the large number of examples available in both classes, we set k = 10 to allow a pertinent similarity analysis. ReliefF can capture local linear and nonlinear interactions among multiple features, depending on the value of k.
- LASSO regression captures the linear relationship between the input feature vector and the target output through linear mapping, by minimising an objective function that combines the mean squared prediction error with regularisation terms. The inclusion of $L_{1}$ regularisation promotes sparsity to effectively eliminate redundant features. Due to the linear nature of the model, coefficients with large absolute values can be interpreted as indicators of strong feature influence. The linear model was designed using 10-fold cross-validation in conjunction with a grid search strategy to tune the regularisation strength, λ, between $3.6\times 10^{-5}$ and 0.36, the best value being λ = 0.0029. Although limited to linear dependencies, LASSO regression can offer insightful information about data.

Feature ranking was used to determine the most informative variables and to build a compact feature vector. After selection, however, the ranks were discarded to avoid introducing bias into the classifier. The model then learns its hierarchy of feature importance directly from the data, using the selected feature subset. In tree-based models, for instance, features that reduce impurity most strongly are implicitly used near the root.

#### 4.4.2. Dimension Reduction Using Feature Vector Transformation

In addition, we investigated methods that transform the original feature vectors into compact, abstract representations. Although these approaches alter the initial attributes, the resulting features might capture more informative patterns relevant to the classification task.

To this end, we employed PCA, which provides linear mapping of the input space. This transformation can remain interpretable, particularly when the dimensionality of the resulting basis is reduced. To mitigate numerical issues arising from the computation of the eigenvalues associated with a large covariance matrix, we also applied PCA separately to 10 disjoint subsets of features and constructed the final reduced feature vector by concatenating the resulting components. The subsets were designed to keep related features together, ensuring that features associated with the same property were not split across different groups, and each original feature is assigned to a group. In all cases, a grid search was performed to determine the threshold for retaining eigenvalues, exploring the range [0.0001, 0.01], which defined the reduced eigenvector basis.

Refined nonlinear transformations of the original attributes into compact feature vectors were obtained using autoencoders. The autoencoders were implemented as neural networks with fully connected layers and hyperbolic tangent activation functions. Each encoder consisted of a single hidden layer, which defined the size of the latent code used later as the input for the classification model. The decoder mirrored this structure, also comprising a single layer, to reconstruct the input. The encoder–decoder network was trained by minimising the mean squared reconstruction error, and only the trained encoder was retained for subsequent feature reduction.

Similar to the PCA-based approach, we also employed a set of autoencoders, each operating on a disjoint subset of the original features. This decomposition reduces the input dimensionality processed by each autoencoder, offering several advantages, most notably, the use of simpler models with fewer trainable parameters, which facilitates easier training. The latent codes generated by all encoders were concatenated to form the final input vector for classification.

The number of neurons in the encoder layer was determined via grid search, considering values between 5 and 500 for the autoencoder trained on the full feature set, and between 5 and 50 for those trained on subsets. The autoencoders working with subsets of features have the same number of neurons in the encoding layer. All models were trained using the ADAM optimiser, with a maximum of 200 epochs and an initial learning rate of 0.001. To prevent overfitting and promote sparsity in the latent representation, training incorporated both $L_{2}$ regularisation (with the associated coefficient equal to 0.001) and sparsification (with the associated coefficient equal to 2).

### 4.5. Classification Models

We selected ensembles of tree-based models capable of handling a large number of features, specifically, Random Forest and XGBoost. These models use a collection of decision trees designed for subsets of samples and features. In Random Forest, uncorrelated trees are built using bagging, whereas XGBoost adds trees iteratively, with each new tree aiming to correct the residual errors of the previous ones. A grid search procedure was applied for hyperparameter tuning to explore various configurations, including variations in the number of trees ($N_{T}$). The goal was to ensure the trade-off between accuracy and generalisation capability. For XGBoost, the experiments were conducted using 80% of the samples and features at each step, with a learning rate of $l_{T}\;=\;0.1$.

In addition, linear logistic regression models using $L_{1}$ regularisation were investigated. These models were chosen for their ability to combine the simplicity and interpretability of linear models with the sparsity-inducing effect of regularisation, which helps diminish the number of active features. At this stage, the variables previously selected via MRMR were introduced into the logistic regression framework, where the $L_{1}$ penalty automatically eliminated less informative features by shrinking their coefficients to zero—serving as a second step of feature selection in case the previous one was insufficient. The regularisation hyperparameter values were determined through grid search, ensuring that the penalisation level preserves a good model’s generalisation ability without compromising discriminative power. This approach also offered additional transparency, as each non-zero coefficient could be directly interpreted as an importance score for the associated feature, thus facilitating the identification of the most relevant biomarkers for toxicity assessment.

### 4.6. Model Evaluation

To evaluate classification performance, we employed metrics such as accuracy and F1 score, and we also visualised the confusion matrices on both training and test data. The same training–testing data partition was used across all experiments to ensure a fair comparison between methods. Furthermore, we used a common random seed for all stochastic techniques to enhance reproducibility.

For certain hyperparameters, grid search was performed end-to-end, considering the entire processing pipeline, including both feature reduction and classification. This involved tuning parameters such as the number of features retained after reduction, $N_{F}$, and classification-related settings, such as the number of trees in ensemble models, $N_{T}$, and the regularisation strength λ for the linear logistic regression model. In the case of MRMR, ReliefF, and LASSO, $N_{F}$ directly specifies the number of top-ranked features selected from the sorted list. For autoencoders, however, $N_{F}$ refers to the total number of neurons in the encoder layers; when using autoencoders on subsets of features, each encoder contains $N_{F}/10$ neurons. For PCA, $N_{F}$ is determined indirectly by setting the eigenvalue threshold $T_{PCA}$, which governs the selection of the eigenvector basis derived from the covariance matrix.

As mentioned in Table 1, the initial experiments combined feature selection methods (MRMR, ReliefF, and LASSO) with previously mentioned classification models (RF, XGBoost, and linear logistic regression). This analysis revealed better results for ensemble models, which were later used in combination with PCA and autoencoders. Finally, we used Wilcoxon signed-rank tests to confirm that the outputs of the classification models, when coupled with different feature reduction techniques, are statistically distinct.

**Figure 7:**
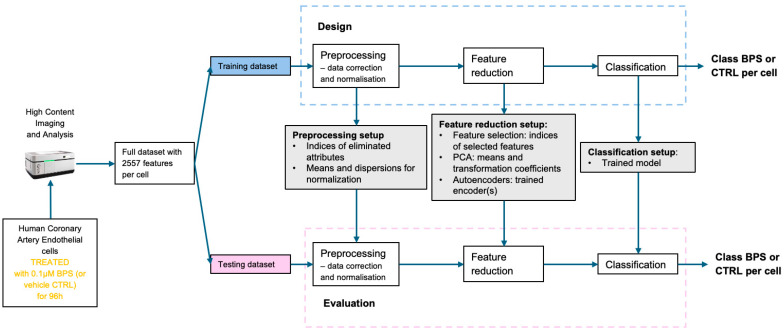
The main steps of data processing for toxicity assessment.

Figure 7 provides an overview of the data flow, illustrating how the entire design is applied to the training dataset to ensure a fair evaluation on the testing set. The attributes acquired from the microscope are first pre-processed for correction and normalisation. Next, the feature-reduction block generates a compact and relevant representation, which is then forwarded to the classification model to determine the final class label, BPS or CTRL. The setup resulting from the training is directly used for testing data, without modifications. This separation ensures a fair evaluation, without the risk of data leakage.

## 5. Conclusions

In summary, our study demonstrates that prolonged low-dose BPS exposure induces subtle yet quantifiable alterations in coronary artery endothelial cells. By using a unified workflow that combines feature reduction with classification, we show that these changes can be detected with high accuracy. Our systematic comparison reveals that accurate predictions can be achieved with computationally efficient models, with XGBoost combined with ReliefF-selected features yielding the best performance. The strong contribution of mitochondrial and nuclear features suggests potential disruption of energy metabolism and genomic regulation as early hallmarks of BPS toxicity. These findings support the perspective that BPS, despite being promoted as a safer BPA alternative, produces measurable vascular effects even at low doses.

By validating robust predictive models, our work bridges cellular toxicology with computational modelling and contributes to a scalable framework for phenotypic toxicology. The analysis combines a dual perspective, a systematic assessment of feature-reduction and classification strategies together with a biological interpretation of resulting patterns, thereby strengthening the value of our findings. This work can be extended by validating the generalisation capabilities of the models across multiple endothelial subtypes and doses to identify patterns of BPS toxicity in a large range of exposure levels, cellular contexts, and phenotypic responses.

## Figures and Tables

**Table 1:** The configurations considered for the experimental analysis.

No.	Feature Reduction	Classification	Hyperparameters Tuned by End-to-End Grid Search ^1^
I	(i) none or	Random Forest or	*N_T_* ∈ {5, 10, 50, 100, 150};
	(ii) MRMR, ReliefF or LASSO	XGBoost	(i) *N_F_* = 2557;
			(ii) *N_F_* ∈ {3, 5, 10, 50, 100}
II	(i) none or	LASSO logistic	*λ* ∈ [3.6 × 10^−5^, 0.36];
	(ii) MRMR	regression	(i) *N_F_* = 2557;
			(ii) *N_F_* ∈ {100, 500, 1000}
III	(i) PCA for all features or	Random Forest or	*N_T_* ∈ {5, 10, 50, 100, 150};
	(ii) PCA applied separately	XGBoost	*T_PCA_* ∈ [0.0001, 0.01]
	to 10 feature subsets		
IV	(i) one AE for all features or	Random Forest or	*N_T_* ∈ {5, 10, 50, 100, 150};
	(ii) 10 AEs for feature subsets	XGBoost	(i): *N_F_* ∈ {5, 10, 50, …, 500};
			(ii): *N_F_* ∈ {50, 100, 200, …, 500}

^1^ Here, hyperparameters are defined as follows: *N_T_* denotes the number of trees used in Random Forest or XGBoost; *N_F_* specifies the number of features retained after feature reduction; *λ* is the regularisation strength used for the linear regression model; *T_PCA_* is the threshold employed by PCA for the eigenvalues of the covariance matrix.

**Table 2:** Experimental results for ensemble models using feature selection.

Trial		Configuration			Training		Testing	
No.	#	Model	Data Reduction	Hyperparameters ^1^	Accuracy [%]	F_1_ Score	Accuracy [%]	F_1_ Score
	1		None	*N_F_* = 2557, *N_T_* = 5	0.99006	0.99060	0.88997	0.89702
	2			*N_F_* = 2557, *N_T_* = 50	1	1	0.92560	0.93054
	3			*N_F_* = 2557, *N_T_* = 150	1	1	0.92911	0.93367
	4			*N_F_* = 3, *N_T_* = 5	0.98047	0.98151	0.87614	0.8825
	5			*N_F_* = 3, *N_T_* = 50	0.99941	0.99944	0.88276	0.8868
	6			*N_F_* = 3, *N_T_* = 150	1	1	0.88218	0.88884
	7			*N_F_* = 50, *N_T_* = 5	0.98648	0.98722	0.9059	0.91145
	8	Random	MRMR	*N_F_* = 50, *N_T_* = 50	0.99991	0.99992	0.92132	0.92620
	9	Forest		*N_F_* = 50, *N_T_* = 150	1	1	0.92171	0.92648
	10			*N_F_* = 100, *N_T_* = 5	0.99148	0.99195	0.91450	0.91931
	11			*N_F_* = 100, *N_T_* = 50	1	1	0.93008	0.93406
	12			*N_F_* = 100, *N_T_* = 150	1	1	0.9322	0.93595
	13			*N_F_* = 3, *N_T_* = 5	0.96411	0.96618	0.87030	0.87815
	14			*N_F_* = 3, *N_T_* = 50	0.98230	0.9833	0.87789	0.88510
	15			*N_F_* = 3, *N_T_* = 150	0.98456	0.98543	0.8788	0.88578
	16			*N_F_* = 50, *N_T_* = 5	0.98956	0.99014	0.9096	0.9148
	17		ReliefF	*N_F_* = 50, *N_T_* = 50	0.99991	0.99992	0.9248	0.92956
	18			*N_F_* = 50, *N_T_* = 150	0.99991	0.99992	0.92560	0.93024
	19			*N_F_* = 100, *N_T_* = 5	0.99574	0.99598	0.91840	0.92307
	20			*N_F_* = 100, *N_T_* = 50	1	1	0.9355	0.93932
	21			*N_F_* = 100, *N_T_* = 150	**1**	**1**	**0.93865**	**0.94223**
	22			*N_F_* = 2557, *N_T_* = 5	0.96636	0.96831	0.92132	0.92289
I	23		None	*N_F_* = 2557, *N_T_* = 50	0.99649	0.99668	0.92717	0.92911
	24			*N_F_* = 2557, *N_T_* = 150	1	1	0.93300	0.93516
	25			*N_F_* = 3, *N_T_* = 5	0.90501	0.91184	0.88802	0.89493
	26			*N_F_* = 3, *N_T_* = 50	0.90920	0.91498	0.88977	0.89626
	27			*N_F_* = 3, *N_T_* = 150	0.92639	0.93089	0.8866	0.89297
	28			*N_F_* = 50, *N_T_* = 5	0.93799	0.94159	0.91158	0.9162
	29		MRMR	*N_F_* = 50, *N_T_* = 50	0.96636	0.9683	0.9248	0.92943
	30			*N_F_* = 50, *N_T_* = 150	0.99816	0.99826	0.92794	0.93225
	31			*N_F_* = 100, *N_T_* = 5	0.95159	0.95441	0.92755	0.93154
	32			*N_F_* = 100, *N_T_* = 50	0.98272	0.98369	0.94274	0.94581
	33			*N_F_* = 100, *N_T_* = 150	0.99991	0.99992	0.94878	0.95144
	34	XGBoost		*N_F_* = 3, *N_T_* = 5	0.90553	0.91164	0.89094	0.89799
	35			*N_F_* = 3, *N_T_* = 50	0.90661	0.91266	0.89152	0.89830
	36			*N_F_* = 3, *N_T_* = 150	0.91479	0.92030	0.88919	0.89580
	37			*N_F_* = 50, *N_T_* = 5	0.93874	0.9422	0.91528	0.9197
	38		ReliefF	*N_F_* = 50, *N_T_* = 50	0.96962	0.97140	0.92950	0.9336
	39			*N_F_* = 50, *N_T_* = 150	0.99908	0.99913	0.93339	0.9370
	40			*N_F_* = 100, *N_T_* = 5	0.95209	0.95494	0.93476	0.93870
	41			*N_F_* = 100, *N_T_* = 50	0.98731	0.98803	0.94897	0.95196
	42			*N_F_* = 100, *N_T_* = 150	**1**	**1**	**0.95637**	**0.95871**
	43		LASSO	*N_F_* = 100, *N_T_* = 5	0.68071	0.70428	0.59600	0.62233
	44			*N_F_* = 100, *N_T_* = 50	0.85390	0.8643	0.62395	0.65313
	45			*N_F_* = 100, *N_T_* = 150	0.96094	0.96326	0.62356	0.65038

^1^ The significance of the hyperparameters is the same as in Table 1. The best results are marked in bold

**Table 3:** Experimental results for the LASSO logistic regression.

Trial		Configuration			Training		Testing	
No.	#	Model	Data Reduction	Hyperparameters ^1^	Accuracy [%]	F_1_ Score	Accuracy [%]	F_1_ Score
II	1	Lasso logistic regression	None	*T_PCA_* = 0.01, *N_T_* = 5	0.77134	0.7828	0.72969	0.7451
	2		MRMR	*T_PCA_* = 0.01, *N_T_* = 50	0.85562	0.8638	0.76299	0.7751
	3			*T_PCA_* = 0.01, *N_T_* = 150	0.91738	0.9223	0.76981	0.7824

^1^ The significance of the hyperparameters is the same as in Table 1.

**Table 4:** Experimental results for models using PCA or autoencoders for feature reduction.

Trial		Configuration			Training		Testing	
No.	#	Model	Data Reduction	Hyperparameters ^1^	Accuracy [%]	F_1_ Score	Accuracy [%]	F_1_ Score
	1		PCA for	*T_PCA_* = 0.01, *N_T_* = 5	0.77134	0.7828	0.72969	0.74513
	2		the full	*T_PCA_* = 0.01, *N_T_* = 50	0.85562	0.8638	0.76299	0.77517
	3		feature set ^2^	*T_PCA_* = 0.01, *N_T_* = 150	0.91738	0.9223	0.76981	0.78240
	4			*T_PCA_* = 0.0001, *N_T_* = 5	0.79320	0.80280	0.7480	0.75974
	5			*T_PCA_* = 0.0001, *N_T_* = 50	0.88183	0.88855	0.7889	0.79933
III	6	XGBoost		*T_PCA_* = 0.0001, *N_T_* = 150	**0.9552**	**0.95780**	**0.8077**	**0.81786**
	7		PCA for	*T_PCA_* = 0.01, *N_T_* = 5	0.74238	0.760158	0.7102	0.72856
	8		10-feature	*T_PCA_* = 0.01, *N_T_* = 50	0.83918	0.84934	0.7507	0.76444
	9		subsets ^3^	*T_PCA_* = 0.01, *N_T_* = 150	0.92773	0.93175	0.7639	0.77590
	10			*T_PCA_* = 0.0001, *N_T_* = 5	0.75431	0.77039	0.71801	0.73509
	11			*T_PCA_* = 0.0001, *N_T_* = 50	0.87440	0.88239	0.77117	0.78577
	12			*T_PCA_* = 0.0001, *N_T_* = 50	0.95193	0.9549	0.78928	0.80255
	1		Autoencoders	*N_F_* = 50, *N_T_* = 5	0.70867	0.73789	0.62317	0.65818
	2		for the full	*N_F_* = 50, *N_T_* = 50	0.85262	0.86367	0.66290	0.69149
	3		feature set	*N_F_* = 50, *N_T_* = 150	0.96177	0.96411	0.67322	0.69830
	4			*N_F_* = 150, *N_T_* = 50	0.75999	0.78182	0.66854	0.70066
	5			*N_F_* = 150, *N_T_* = 150	0.91571	0.92143	0.71626	0.73780
	6			*N_F_* = 150, *N_T_* = 50	0.99432	0.9946	0.74508	0.76134
IV	7	XGBoost		*N_F_* = 500, *N_T_* = 5	0.8493	0.85630	0.79785	0.80777
	8			*N_F_* = 500, *N_T_* = 50	0.96945	0.97109	0.84420	0.85223
	9			*N_F_* = 500, *N_T_* = 150	1	1	0.87186	0.87855
	10		Autoencoders	*N_F_* = 50, *N_T_* = 5	0.68405	0.71318	0.61226	0.64965
	11		for 10-feature	*N_F_* = 50, *N_T_* = 50	0.80347	0.82007	0.62512	0.65911
	12		subsets	*N_F_* = 50, *N_T_* = 150	0.92606	0.93097	0.62395	0.65238
	13			*N_F_* = 150, *N_T_* = 5	0.78694	0.80429	0.71840	0.74288
	14			*N_F_* = 150, *N_T_* = 50	0.93123	0.93560	0.79006	0.8047
	15			*N_F_* = 150, *N_T_* = 150	0.99290	0.99329	0.81324	0.82547
	16			*N_F_* = 500, *N_T_* = 5	0.92531	0.93024	0.89873	0.90510
	17			*N_F_* = 500, *N_T_* = 50	0.98397	0.98488	0.9281	0.93250
	18			*N_F_* = 500, *N_T_* = 150	**1**	**1**	**0.93534**	**0.93910**

^1^ The significance of the hyperparameters is the same as in Table 1. ^2^ For *T_PCA_* = 0.01, the number of features after reduction results *N_F_* = 599, while for *T_PCA_* = 0.0001, the reduced feature vector includes *N_F_* = 2053 elements. ^3^ For *T_PCA_* = 0.01, the number of features after reduction results *N_F_* = 1690, while for *T_PCA_* = 0.0001, the reduced feature vector includes *N_F_* = 2474 elements. The best results are marked in bold.

## Data Availability

The data presented in this study are available on request from the corresponding authors.

## Abbreviations

The following abbreviations are used in this manuscript:

AE	Autoencoder
BPS	Bisphenol S
BPA	Bisphenol A
EDC	Endocrine-disrupting Chemical
HCI	High-content microscopy
LASSO	Least Absolute Shrinkage and Selection Operator
ML	Machine Learning
MI	Mutual Information
MRMR	Minimum Redundancy Maximum Relevance
OOB	Out-of-Bag
PCA	Principal Component Analysis
RF	Random Forest
XGBoost	Extreme Gradient Boosting
